# Ethidium bromide interactions with DNA: an exploration of a classic DNA–ligand complex with unbiased molecular dynamics simulations

**DOI:** 10.1093/nar/gkab143

**Published:** 2021-03-25

**Authors:** Rodrigo Galindo-Murillo, Thomas E Cheatham

**Affiliations:** Department of Medicinal Chemistry, College of Pharmacy, University of Utah, 2000 East 30 South Skaggs 306, Salt Lake City, UT 84112, USA; Department of Medicinal Chemistry, College of Pharmacy, University of Utah, 2000 East 30 South Skaggs 306, Salt Lake City, UT 84112, USA

## Abstract

Visualization of double stranded DNA in gels with the binding of the fluorescent dye ethidium bromide has been a basic experimental technique in any molecular biology laboratory for >40 years. The interaction between ethidium and double stranded DNA has been observed to be an intercalation between base pairs with strong experimental evidence. This presents a unique opportunity for computational chemistry and biomolecular simulation techniques to benchmark and assess their models in order to see if the theory can reproduce experiments and ultimately provide new insights. We present molecular dynamics simulations of the interaction of ethidium with two different double stranded DNA models. The first model system is the classic sequence d(CGCGAATTCGCG)_2_ also known as the Drew–Dickerson dodecamer. We found that the ethidium ligand binds mainly stacked on, or intercalated between, the terminal base pairs of the DNA with little to no interaction with the inner base pairs. As the intercalation at the terminal CpG steps is relatively rapid, the resultant DNA unwinding, rigidification, and increased stability of the internal base pair steps inhibits further intercalation. In order to reduce these interactions and to provide a larger groove space, a second 18-mer DNA duplex system with the sequence d(GCATGAACGAACGAACGC) was tested. We computed molecular dynamics simulations for 20 independent replicas with this sequence, each with ∼27 μs of sampling time. Results show several spontaneous intercalation and base-pair eversion events that are consistent with experimental observations. The present work suggests that extended MD simulations with modern DNA force fields and optimized simulation codes are allowing the ability to reproduce unbiased intercalation events that we were not able to previously reach due to limits in computing power and the lack of extensively tested force fields and analysis tools.

## Graphical Abstract

**Graphical Abstract:**
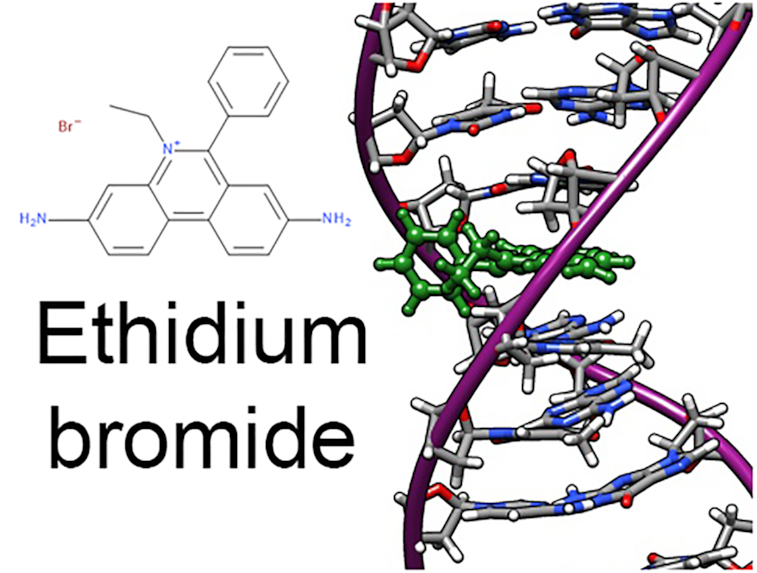
Ethidium intercalation as captured by unbiased molecular dynamics simulations.

## INTRODUCTION

**Figure 1.**
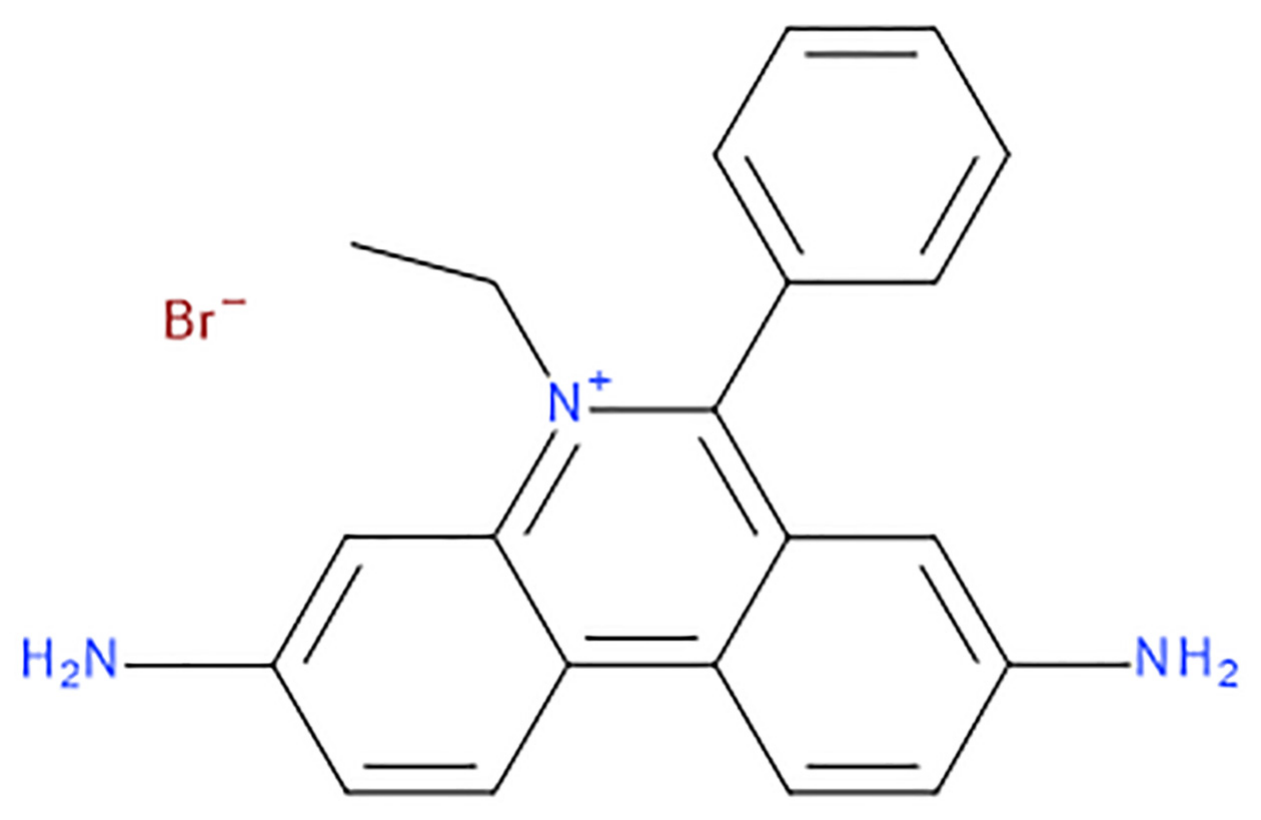
Chemical structure of ethidium bromide, IUPAC name: 3,8-diamino-5-ethyl-6-phenylphenanthridinium bromide.

The ethidium bromide compound (EtBr, Figure 1) started its application in chemistry and biology as a treatment for certain infections, mainly parasites (1,2). Over the years, this useful fluorescent dye has become a necessary chemical compound in molecular biology labs across the world to help in the visualization of nucleic acids. Ethidium was incorporated for the first time in 1969 as a staining agent in electrophoresis gel analysis due to a broken-down centrifuge, as explained in a very enjoyable manner by Piet Borst (3). When trying to separate the circular, nicked and linear forms of vertebrate's mitochondrial DNA, their ultracentrifuge stopped working. They then switched to electrophoresis in agar gels to separate the different shapes of the DNA they extracted, with the problem of not being able to visualize the running bands in the gel. The group had extensive experience running CsCl-ethidium equilibrium gradients to purify DNA, which showed bright orange bands. This in turn seeded the idea to stain the gels with ethidium after the electrophoretic run. As Borst explains, they never went back into using their analytical ultracentrifuge methodology and with their reported technique, they set the origin for ethidium-agarose electrophoresis.

During the early 1960s, experimental and spectroscopic work showed direct evidence of a strong interaction between planar acridine derivatives (e.g. proflavine, daunorubicin etc.) and nucleic acids which led to speculation about the possible structural binding mode of these molecules with DNA. The binding constants measured by several research groups were too strong to consider only an electrostatic mode of interaction; another binding mode was needed. Lerman proposed in 1961 (4) that these small molecules should intercalate between adjacent nucleobases causing the DNA to unwind and extend the deoxyribose-phosphate backbone to make room for the ligand to insert. Some years later in 1966, the concept was updated by Pritchard and collaborators (5) proposing that the two bases forming the intercalation pocket align with the aromatic part of the intercalated ligand (ethidium bromide, proflavine, 9-amino-tetrahydroacridine) to form strong stacking interactions, thus explaining the stable complex observed through experiments.

It was not until 1975 that the first structural evidence of the intercalation binding mode between the ethidium ligand with DNA nucleobases was published by Henry M. Sobell and collaborators (6). The work from the Sobell lab provided the first experimental evidence of the intercalation of aromatic amino acridines and phenanthridinium molecules between DNA bases. His combined efforts with multiple collaborators produced a collection of ten articles titled: ‘Visualization of drug-nucleic acid interactions at atomic resolution’ (7–16). They report on their work using X-ray crystallography of the complex between ethidium, 9-aminoacridine, proflavine, acridine orange, ellipticine and *N*,*N*-dimethyl proflavine with several dinucleoside monophosphates, mainly 5-Iodouridylyl(3′–5′)adenosine, 5-iodocytidylyl(3′–5′)guanosine, uridylyl(3′-5′)adenosine, cytidylyl(3′-5′) guanosine and deoxy cytidylyl(3′-5′)deoxyguanosine.

In the following 30 years, multiple research articles using dozens of analytical techniques were used to study every possible physical aspect of the interaction of EtBr with nucleic acids. From the earliest experiments, there was evidence that the ethidium molecule binds strongly to both DNA and RNA sites with one molecule bound for every 4 or 5 bp steps under saturating conditions. This same study is perhaps the first proof of a drug-concentration-dependent multi-mode binding between EtBr and nucleic acids, and the complex formation was reversible (17). In these earlier studies, no sequence specificity was detected since most of the DNA was obtained from bulk methods using rat liver, calf thymus or bacterial sources. A brief summary of the main physical observations of the EtBr–DNA complex is presented in Table 1.

**Table 1.** A condensed list of the main physical characteristics obtained from the literature about the EtBr–DNA complex

• The main binding modes include intercalation between base-pairs, semi-intercalation, and electrostatic interaction with DNA phosphate backbone (18–22).
• The binding kinetics of the ethidium–DNA complex, followed using spectroscopy, show a two-stage binding process. The first step is a fast-diffusion of the ligand throughout the DNA polymer, followed by the intercalation event which occurs on the millisecond time range (23–25).
• Intercalation events of EtBr produces negative unwinding of the helix to a value of 26° while increasing the distance between adjacent base pairs (26–29).
• Intercalation of EtBr to the DNA duplex can occur from both the minor and major grooves, however it is very weakly bound when from the major groove. The preference is highly dependent on the length of the DNA, EtBr concentration, the type of included salt, salt concentration, pH and solvent type (17,19,30–32).
• Kinetic analysis of the formation of the EtBr-DNA complex is also highly dependent in the conditions used. For example, an association constant value for poly[d(A–T)] has been reported to be 17 × 10^6^ M^−1^ s^−1^, for poly[d(G-C)] a value of 13 × 10^6^ M^−1^ s^−1^ and for poly(A-U) a value of 5.9 × 10^4^ M^−1^ s^−1^ Similar values within that range have been calculated for multiple sequences and systems using physical methods (33,34) and with quantum mechanical calculations (35) and molecular dynamics simulations that suggest a slight TA sequence specificity.
• Interactions of ethidium with DNA duplexes increases the melting temperature with increased concentration of the ligand (36).
• NMR spectroscopy studies of the EtBr–DNA complex with the triphosphate d(GCGC) and d(CGCG) show a preference of intercalation in the CpG-site from the minor-groove (37). Similar studies with the sequences 5′d(TGCA) show intercalation from the minor-groove in the T–G site and studies with d(ACGT) is also from the minor-groove in the CpG-site (38). With the 8-mer d(GACATCTC) the intercalation is also observed from the minor groove in the CpA-site (39). Major groove binding pathways have been suggested by modeling (40,41), however they have not been seen experimentally likely due to the very weak interaction with DNA when bound in this manner.

It is critical for the computational chemist to test, benchmark and thoroughly assess the methods and practices that we use to construct models, sample dynamical processes, and to measure thermodynamic properties. The ethidium–DNA complex represents a prototypical well studied experimental system to compare small molecule interactions with nucleic acids and to benchmark MD simulations and compare with experimental structures. Even though the ethidium–DNA system has been known for >60 years and has been widely studied by a dozen or more experimental techniques, only a handful of published articles using computational chemistry tools have focused on this system.

Early in the pre-molecular dynamics (MD) simulation days of the theoretical modeling of nucleic acids, ethidium–DNA duplex complexes were studied by molecular mechanics energy refinement by the Kollman group and by normal mode calculations by the Case group (with AMBER 2.0 noting that AMBER 20 was released in 2020), showing the ability to capture the structural implications of increased helix length, unwinding and significant lowering of the mobility of base pairs adjacent to the drug (42,43). Further studies of the ethidium–DNA-duplex complex through MD simulations have been somewhat sparse due to the lack of extended sampling time and the slow timescale for interaction (40,44). The article by Monaco and collaborators perhaps is the first robust MD simulation study of the interaction between ethidium and the 10-mer d(AGGATGCCTG) (40,41), although a rather short (0.5 ns) amount of sampling time is presented for each of the 10 complexes studied. The authors show an externally bound complex with the ethidium molecule within the major groove of the DNA, forming hydrogen bond interactions with the GATG site. In one of their unbiased MD simulations, a very surprising, fast (occurring over ∼3 ps), and very early near the start of the MD simulation intercalation event through the major grove of the DNA was observed. As the authors note, the observation was not reproducible and the authors state that the observation ‘could be an artefactual run, representing a non-physical system’. As the intercalation event was observed very early in the simulation, and so fast, we speculate it was an artefact of the initial ethidium placement. The work of Fresch and Remacle show MD simulations using the AMBER set of tools and force fields (parm99/bsc0) to explore the structure and energetics of the ethidium molecule binding to a 12-mer with sequence d(GGTAAATTTAGG) (45). Their test systems consisted of a 1:1 DNA–ethidium complex, manually inserting the ethidium molecule in the intercalating site (ApA step) with the ligand both from the major and minor grooves and three ethidium molecules inserted in three different base-steps separated by one drug-free site. More recent MD approaches to this system include simulations investigating the enhanced mechanical stability when ethidium is bound and pulled free in un-converged steered MD simulations (46) and also MD simulation of ethidium placed into DNA mini-circles which helps explain increases in DNA translocation times as the ethidium concentration increases (47).

The limited number of publications was mainly due to lack of computer power and the lack of sufficiently reliable force fields for small molecules and nucleic acids. The force fields have improved, and in 2019 with the use of GPU technology, we were able to easily reach microsecond timescales within a week for systems of ∼40–50 000 atoms (48–52). Also, by calculating MD simulations on multiple independent copies, we increase sampling and statistical validation (53) to ultimately get closer to convergence. In this work, we present extensive MD simulations between ethidium bromide (Figure 1) with double stranded DNA in order to detect the intercalation or minor groove binding events without any bias or restraint.

## METHODOLOGY

**Figure 2.**
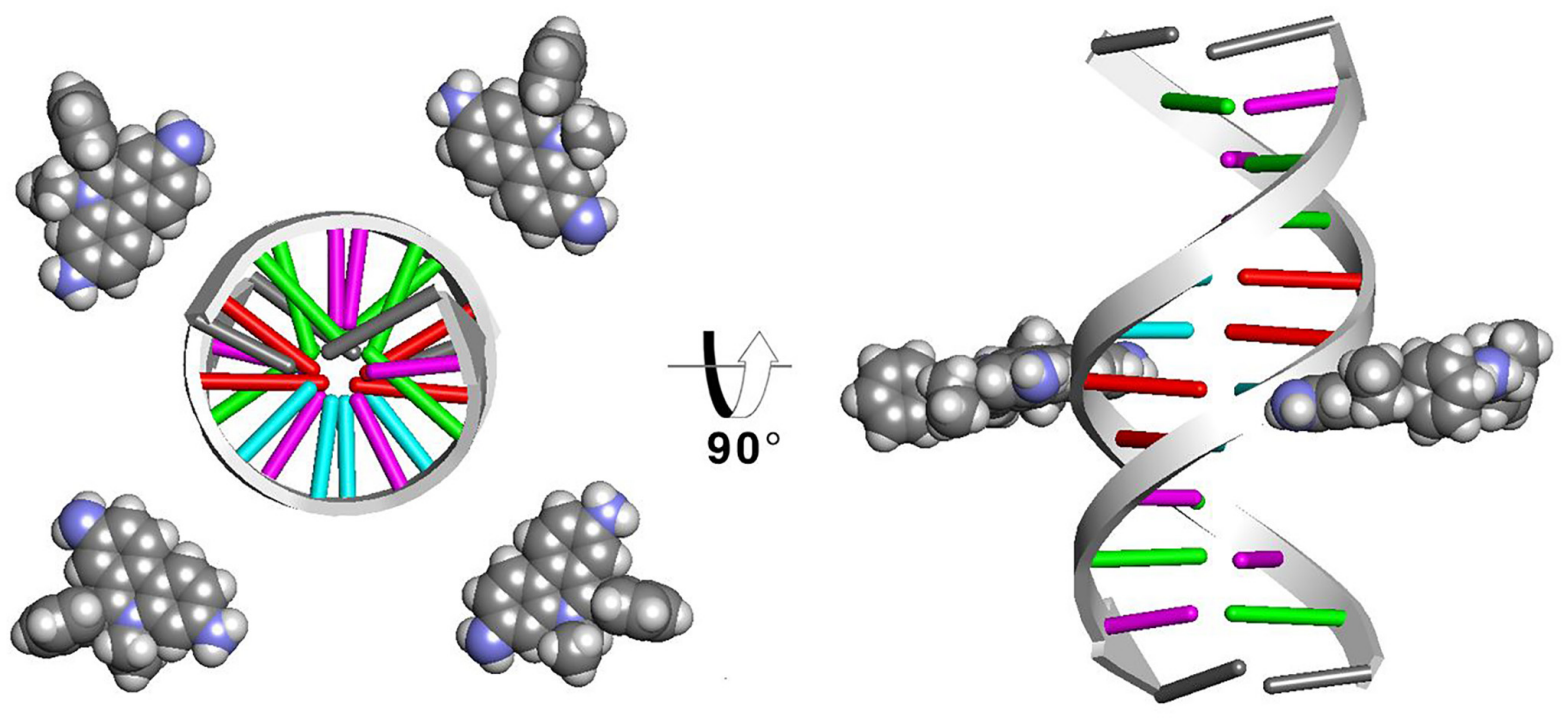
Starting structure for the DDD system (GAAC is similar). Ethidium molecules are shown as spheres around the DNA at a distance of no less than 15 Å from the helical axis. Red is adenine, purple is cytosine, green is guanine and cyan is thymine (with the termini gray).

The starting model for the unbiased MD simulations consisted of four ethidium ligands manually placed around the DNA duplex at a distance of no less than 15 Å from the helical axis (Figure 2). The ethidium molecule atom types were represented by the General Amber Force Field [GAFF (54)], and the charges were computed using the RESP methodology (55) and assigned with antechamber using the Hartree-Fock level of theory with a 6–31* basis set (noting the parameters are reported in a mol2 file in the Supporting Information). The Drew-Dickerson DNA sequence d(CGCGAATTCGCG)_2_ was described by the parm99 (56) AMBER force field with the bsc0 (57) modifications. The system was solvated using the TIP3P water model (58) in a truncated octahedral periodic box and SHAKE was applied to the hydrogens (59). Sodium counter ions to neutralize the charge were added and four bromine ions to represent the ethidium bromide salt. An excess of NaCl was added to reach ∼200 mM concentration. Ions were described by the Joung–Cheatham model for TIP3P (60). Initial minimization was performed for 5000 steps applying a harmonic positional restraint of 10 kcal/mol·Å^2^ to the DNA duplex (with no restraints on the ethidium molecules, water and ions). Further equilibration was performed in a series of steps gradually reducing the restraints in 8 different steps, each ∼5000 ps. Final equilibration was performed for 1000 ps with no restraints in an NTP ensemble using Langevin dynamics (61) (gamma = 1.0) for temperature control at 300 K and the Berendsen barostat for pressure scaling. Long range electrostatics with a cut-off of 9 Å were calculated using the particle-mesh Ewald method (62) using default parameters. Production simulations were run using three independent copies, each independent copy with a sampling time of >93 μs (for a total 279 μs combined) using the pmemd.cuda engine with a 2 fs integration time step (48). The second system tested has the sequence d(GCACGAACGAACGAACGC) and was selected from the ABC series (63,64). This sequence has been used previously to study the convergence and reproducibility in extended AMBER molecular dynamics simulations (51,52). In this case, 20 independent MD simulations were run, each for ∼27 μs of sampling time. Similar to the DDD simulations, four molecules of ethidium were included and manually placed no less than 10 Å from the DNA helical center (Figure 2). The same minimization, equilibration and production protocols described previously were used. The updated OL15 AMBER force field was employed (65–67) instead of the older bsc0 parameters. The same water and ion model was used (final concentration of ∼200 mM) as for the DDD simulation. Hydrogen mass repartitioning on the solute atoms allowed the use of a 4 fs time step for the production simulations (68). Distribution around the DNA duplexes of the ethidium ligand was performed using curvilinear helicoidal coordinates analysis as implemented in the ‘Canion’ program (69). This analysis is based on the instantaneous measure of the DNA helical axis, which was obtained by the Curves+ software (70). Atomic population density grids for the ethidium molecules were calculated using CPPTRAJ for each of the ethidium ligands. First, water and ions were stripped creating an ‘in vacuo’ trajectory and the DNA was aligned into a common reference frame by a root-mean-squared deviation fit to the average structure. With this trajectory, an X-PLOR density map file is generated and is visualized with VMD (71). Biased simulations using the umbrella sampling methodology were additionally performed with the pmemd.MPI program using distance between the center of mass of the selected base-pairs and the center of mass of the ethidium ligand as the reaction coordinate. In total, 100 windows were computed with 0.2 Å increments. A potential of mean force curve was calculated using the weighed histogram analysis methodology (72) as implemented in the WHAM code (A. Grossfield, 2003).

Quantum mechanics calculations and ethidium molecule set-up was performed by the 09.D01 version of Gaussian 16 C.01 revision and Gaussview (Gaussian Inc). Simulations were performed using AMBER14 and AMBER16 (73) codes; and trajectory analysis was performed with the development version of CPPTRAJ (4.12.2 GitHub) (74,75). Energy analysis was performed using the MM-GBSA (76,77) approach with the MMPBSA.py tool (78) available in AmberTools16.

## RESULTS AND DISCUSSION

Overall, the DNA duplex remains stable in the B-DNA conformation for the entire length of the sampled trajectories for all of the models. Fraying events at the termini base-pairs are commonly observed (51,52) and the central base pairs show expected DNA breathing dynamics for a Watson–Crick duplex.

### The Drew–Dickerson dodecamer captures EtBr intercalation at the terminal base-pairs only

**Figure 3.**
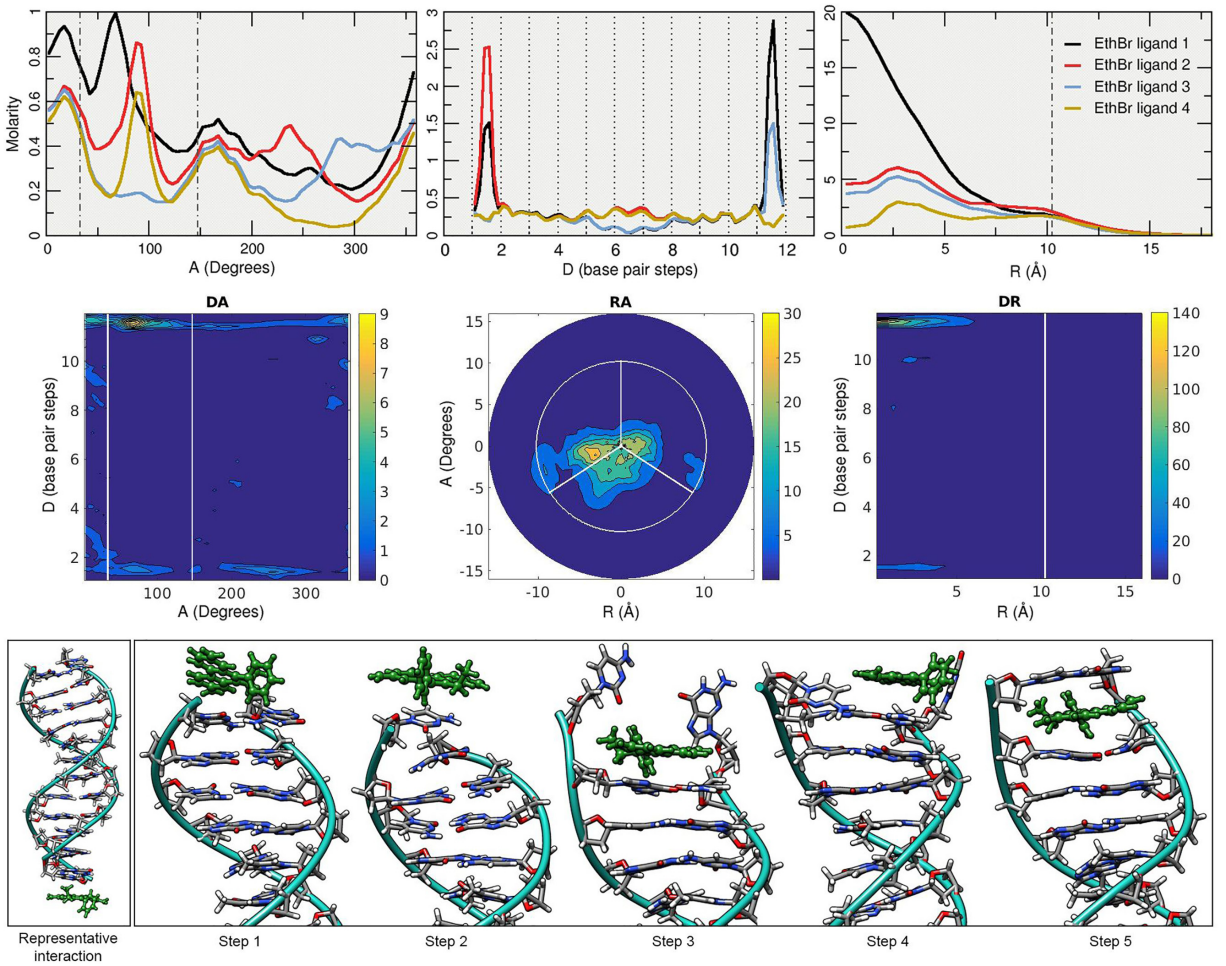
Top row: 1D A, D and R plots of the ethidium ligands. The short distance in the A plot between the dotted lines represents the minor groove using the C1′ atoms angular position. The dashed line in the R plot represents the radial position of the phosphorous atoms; values are based on the Canion publication (69). Each line of the top plots represents the analysis performed in each of the four ethidium ligands. The full trajectory information with an offset of 10 frames was used for the analysis. Middle row: 2D representations for the curvilinear helicoidal information based on the ethidium ligand 1 only. Bottom row, left: selected representative interaction extracted from one of the simulations. Bottom row, right: ethidium intercalation process at the CpG base pair steps at the 5′/3′ ends of the DDD depicted by selected snapshots from the trajectory data.

To study the binding modes of the classic dye ethidium with DNA through MD simulations, we employed the prototypical Drew–Dickerson dodecamer system referred to here as DDD. As depicted in Figure 2, the simulations started with the DNA duplex surrounded by four EtBr molecules (the solvent and ions are not shown). After >93 μs of combined sampling using the three independent MD simulation copies with different initial conditions, the trajectories were aggregated and analyzed. To study the overall distribution density of the EtBr molecules among the DNA duplex, we employed the curvilinear helicoidal coordinates approach centered on the helical axis of the DNA. Briefly, the positions of the ethidium ligands are determined with respect to the helical axis, which allows us to: (i) measure the radial distribution of the ligands (cylindrical distribution function or angular *A* plot), (ii) measure the distribution along the helical axis (distance *D* plot) and (iii) measure the distance from the helical axis towards the bulk of the solvent (radial distance *R* plot, refer to Figure 1 of the original Canion publication (69)). This method allows the calculation of time-averaged populations among the DNA duplexes for each of the four EtBr ligands, and this provides insight into the interaction of these two molecules over the extensive amount of sampling data provided by the MD simulations. The angular *A* plot (top row, Figure 3) depicts the ethidium ligand accumulation within the minor groove region to an extent, and accumulation that explores other values that do not clearly correspond within the minor groove with no discernable trend. When we consider the distance *D* plot (center plot, top row Figure 3), two regions that show strong molarity peaks at both the terminal base pairs of the DNA duplex with close to zero concentration of the ligands within the central base pair region. The radial distance *R* plot shows accumulation in regions below ∼6 Å, and beyond the phosphorous atom radius (indicated by the dashed vertical line) the concentration of ligands decays rapidly. Ethidium ligand 1 (black line) shows a ∼20 molar concentration at 0 Å, which means that most of the ligand spends time at zero distance from the helical axis. This is only possible if the ligand is either in intercalation between the bases or stacked at the ends of the DNA duplex. In combination with the distance *D* plot, we can see high molarity values between the CpG base pair steps at both ends of the chain for ethidium ligand 1 (black line) which suggests that ethidium ligand 1 binds ∼66% of the sampled time between the terminal base-pairs at the 3′/5′ ends and 33% stacked at the 3′/5′ ends of the duplex. Similar behavior is observed for ethidium ligands two to four.

Further refinement of the analysis can be done by 2D representations of the curvilinear helicoidal information (middle row, Figure 3). Combining the angular *A* with the distance *D* plots (*DA*) we can confirm that the ethidium ligand 1 occupies primarily both ends of the DNA duplex with little to no presence in the inner base-pairs. The *RA* and *DR* plots shows the ethidium ligand 1 positioned mainly at the center of the helical axis, consistent with the ligand binding in either intercalation or stacking interactions at the DNA termini. Visual examination of the trajectories confirms the long-lived presence of the ethidium ligands in two main configurations: stacked on the terminal base-pairs or intercalated at the terminal base-pair step. A representative structure of the stacking interaction at the 3′→5′ is depicted in Figure 3 (bottom left). As mentioned, the ethidium ligand stacks in the terminal base-pair and rotates freely with the DNA helical center as the center point. This rotational variation is confirmed by the *A* plot (Figure 3, top left) which represents the angular position with respect to the helical axis. The intercalation process that occurs on both sides of the terminal base-pairs is depicted with molecular graphics at the bottom of Figure 3. Briefly, the ethidium ligand is in a stacking position above the CG base pair (step 1), breathing and fraying motions of the base pair, which are commonly observed (51,52,79), rupture the Watson-Crick pairing (step 2) allowing for the ethidium ligand to move inside the cavity and form a stacking interaction with the second C/G base-pair (step 3). The frayed nucleotides interact with solvent and ions (step 4) until it reforms the WC pairing trapping the ethidium ligand in an intercalated position (step 5). Binding energy for the representative interaction of the ethidium stacked in the terminal base-pairs corresponds to a value of –14.7 (±3.2) kcal/mol, whereas the intercalation interaction is –34.6 kcal/mol (±2.5). Both values were extracted from an average of the MM-GBSA calculations on 100 frames, noting that this method tends to over-estimate the binding affinity somewhat and is typically better for relative free energy comparisons. Although ethidium can in principle bind to sites every 4–5 base pairs at saturation, the simulations do not show intercalation at the central AT step. This could be due to the kinetics and enhanced thermal stability or simply this was not observed because the simulations were not long enough. However, as the intercalation at the ends of the helix occurs more rapidly than with internal base pairs, and these interactions stabilize and rigidify the helix, this could in principle inhibit intercalation at the central steps. Although our speculation is that the end base pair step inhibits internal base pair intercalation, it could possibly also relate to sequence in the AATT A-track which tends towards a narrower minor groove.

### The extended 18-mer sequence allows greater exploration of additional ethidium binding modes

Interesting insights were provided by using the 12-mer sequence d(CGCGAATTCGCG), although it is clear that this particular sequence biased the ethidium-DNA interaction towards two modes (i.e. stacking on the end base-pairs or intercalation at the terminal CpG base pair step). In order to investigate if a longer sequence would provide additional binding modes, we used an 18-mer with the sequence d(GCACGAACGAACGAACGC) from the set of ABC tetramers (63,64) that our lab has extensively studied (51,52), referred to from now on as GAAC. Even though we used an older version of the force field for the DDD example (bsc0), we do not expect to observe any significant differences with regards to the interactions between the ethidium molecule and the DNA due to the update in the force field.

**Figure 4.**
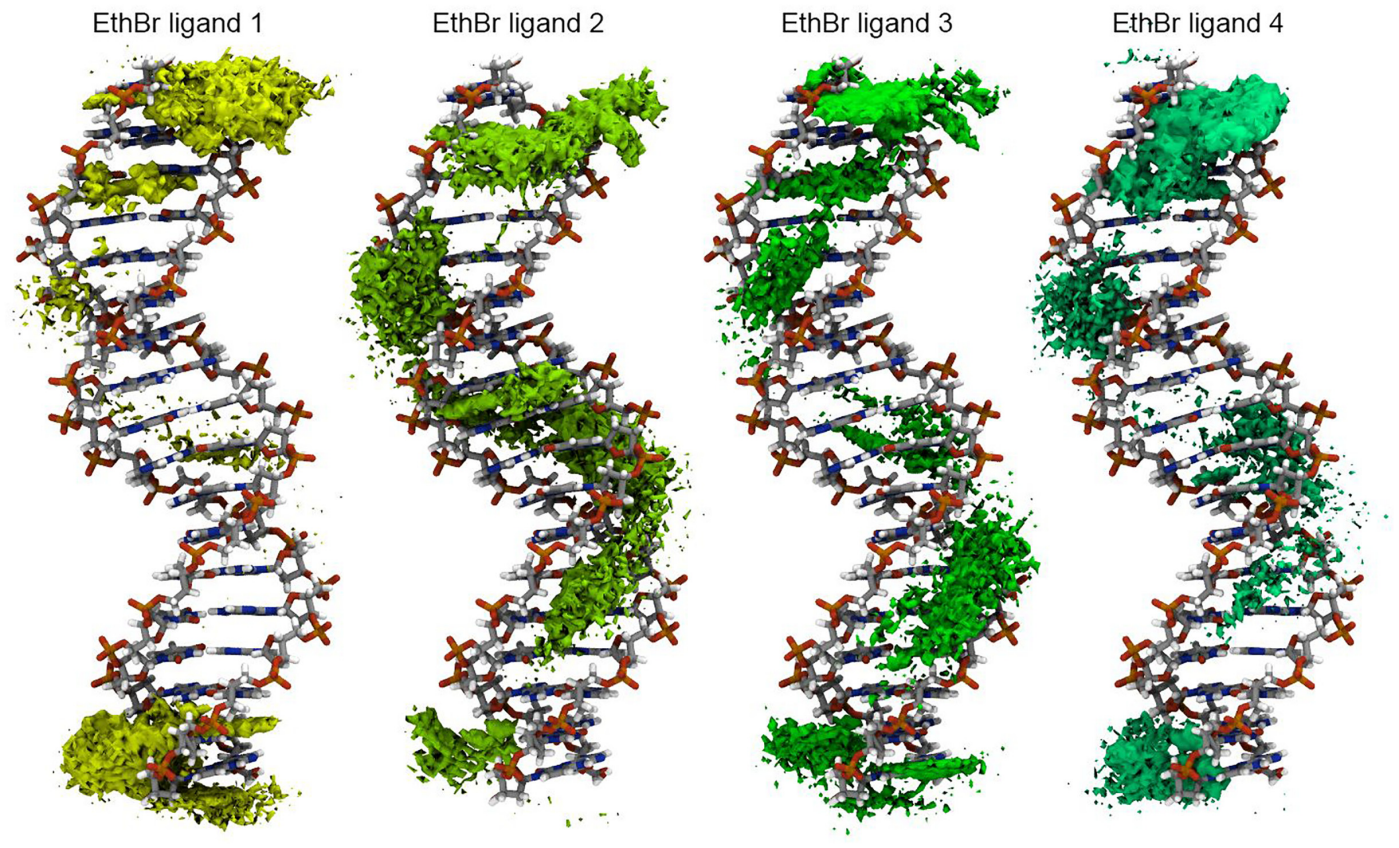
Atomic density population histograms for each of the ethidium ligands taking into account every 10th frame from all of the trajectory information (using the 20 independent copies). The GAAC duplex structure shown is a 10 μs simulation average structure without ethidium present, which does not have the characteristic DNA deformations seen in previous studies of ethidium interaction with duplexes or this work Figures 3 and 6, and Supplementary Figure S1 and Figure S2.

Given the extensive amount of data generated, as a first approach, a grid-based atomic density population histogram for each of the four ethidium ligands was computed and visualized (Figure 4). This enables a quick visual assessment of the average interactions of the ethidium molecules within the GAAC sequence. As observed with the DDD, there is an accumulation of the ligand at both ends of the duplex, although in this case, it is clear that additional binding modes appear. Little to no ligand accumulation density appears in the major groove, suggesting that interactions through the major groove are less likely and/or very short lived. Accumulation data of the ethidium ligands is observed throughout the minor groove and intercalation events both in the base-pairs at the end of the GAAC and in the central regions are observed. Remembering that we have a simulation with four free binders, ethidium ligand 2, for example, shows a clear intercalation event in the central base-pairs of the GAAC duplex and ligands 1, 3 and 4 present a similar intercalation density close to the terminal base-pairs, noting that since the shown reference DNA is an average structure without ethidium present, the DNA shown does not have the characteristic DNA deformations seen in previous studies of ethidium interaction with duplexes (45).

**Figure 5.**
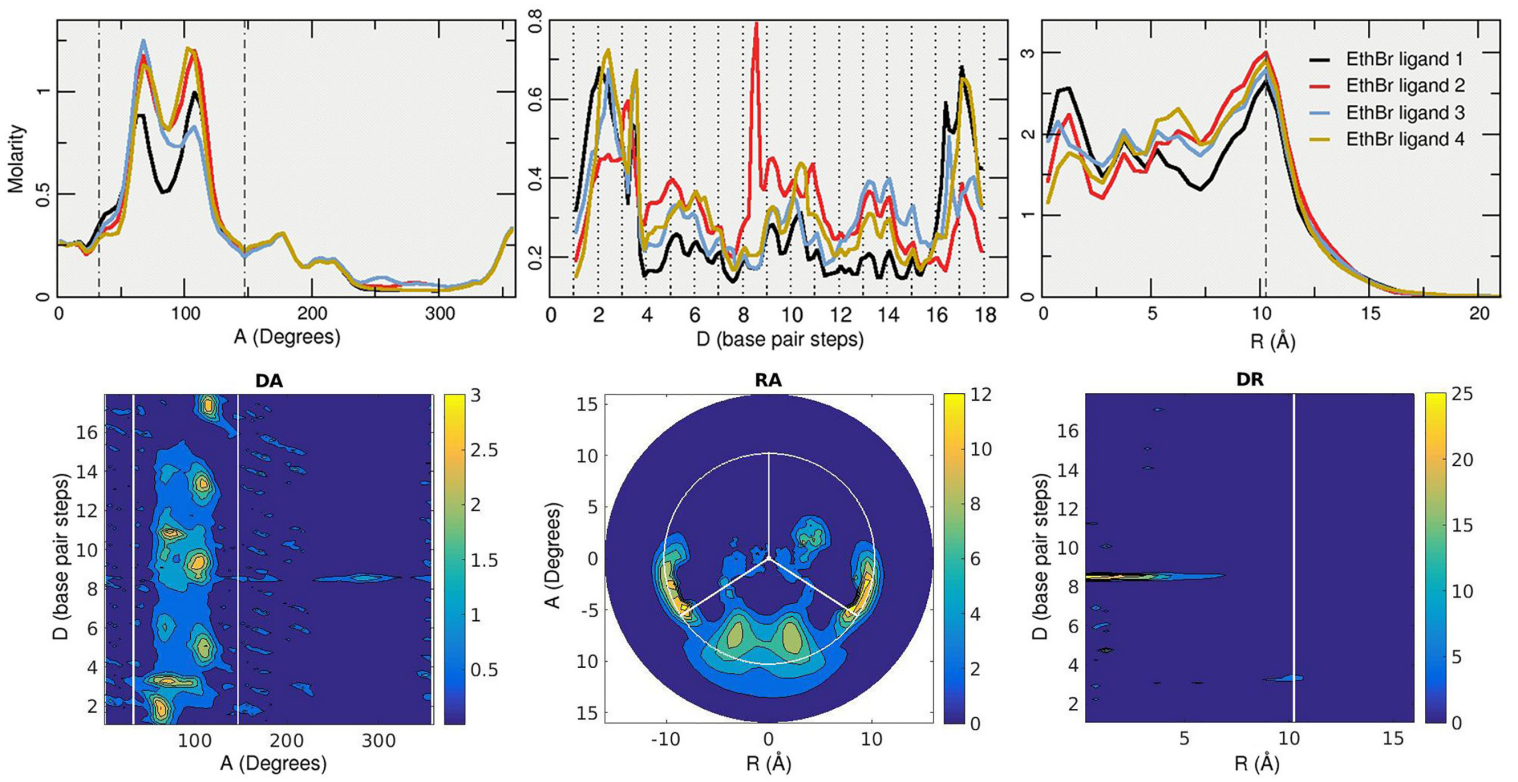
Top row: 1D A, D and R plots of the ethidium ligands. The short distance in the A plot between the dotted lines represents the minor groove using the C1′ atoms angular position. The dashed line in the R plot represents the radial position of the phosphorous atoms. Bottom row: 2D representations for the ethidium ligand 2 set of trajectories (red line on the 1D plots). Molarity increases from blue to yellow.

The curvilinear helicoidal coordinate analysis provides further detail on the coarse image rendered by the density histograms. The angular *A* plot (top row, Figure 5) shows elevated concentration of the ethidium ligands within the minor groove for all four molecules. The bimodal accumulation present in the minor groove corresponds to the ligand within the minor groove (accumulation at ∼68°) and intercalation between the base pairs (accumulation at ∼105°). These two modes of interaction are observed also in the umbrella sampling simulations (see next section, Figure 7). The axis distance *D* plot shows multiple accumulation points throughout the GAAC duplex. In contrast with the same plot from the DDD sequence simulations, which show little to no concentration within the central inner base-pairs, the GAAC simulations shows multiple contact points that translates to the ethidium ligands exploring the minor groove. We know that the ligands are within the minor grove since the *A* plot shows the majority of the molarity within that region. Of special interest from the *D* plot is ethidium ligand 2 (red line) that presents an ∼8 molarity peak between the C8pG9 base-pair step, which is also observed in the previous grid histogram analysis. Radial plot *R* shows similar molarity values of ∼1–2.5 at very close distance of the GAAC helical axis (intercalation and stacking events) and higher values of ∼2.5–3 molarity close to a value of 10 Å which corresponds to the edge of the GAAC duplex from the minor groove region.

The 2D plots complement the 1D data (bottom row, Figure 5). In this case, the data is from the ethidium ligand 2 that presents the sharp intercalation event in the C8pG9 region as can be seen from the *DA* and *DR* plots. High accumulation within the minor groove is visible from the data presented in the *DA* and *RA* plots and to some extent the interaction with the terminal base-pairs. All the trajectories were visually inspected to extract the most representative binding modes found between the ethidium molecule and the GAAC sequence. As expected with the amount of sampling calculated in this work, the observed binding modes can be broadly classified in five states: stacking on the terminal base pair, minor groove binding, intercalation through base-pair eversion, intercalation from the minor groove and intercalation from the major groove. A representative structure of each binding mode is depicted in Figure 5. After stacking interactions at both ends of the GAAC sequence, the most commonly observed binding mode corresponds to the phenanthridine moiety of the ethidium ligand in direct contact within the minor groove of the DNA, with the 6-phenyl ring towards the solvent. In this binding mode, the ligand freely explores the length of the minor groove interacting with the ApA regions as observed in the 2D *RA* plot (Figure 5, notice the three regions of high molarity that correspond to the three GAAC repeats) and the population density plot.

**Figure 6.**
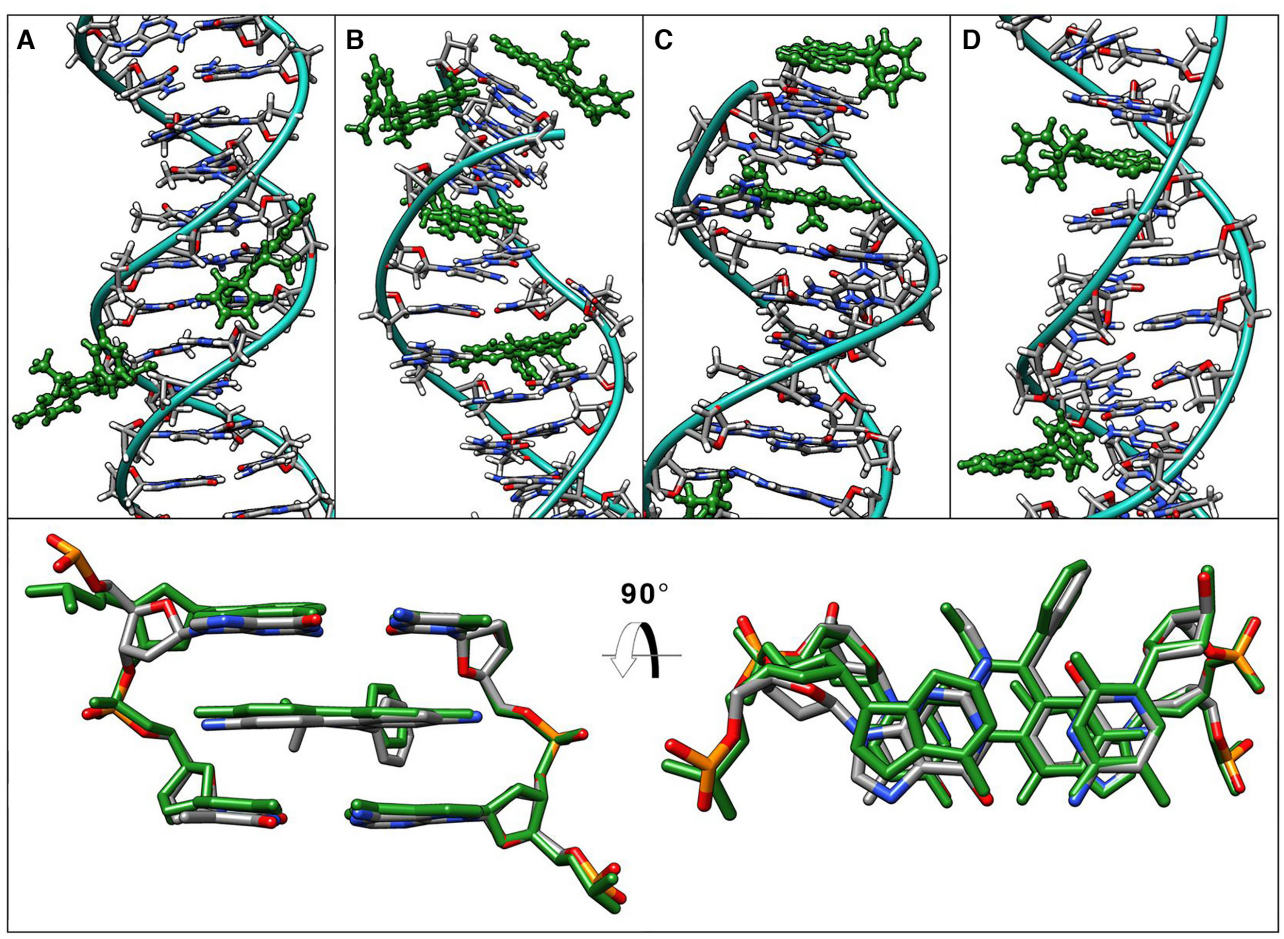
Top panel: selected representative binding modes of the ethidium ligand with the GAAC sequence. (**A**) Minor groove binding, (**B**) intercalation interaction between the ApC step and a base-pair eversion insertion mode in the A6:T31 step, (**C**) stacking on the terminal base-pairs and insertion mode on the A3:T34 position and (**D**) major groove intercalation between the ApA step and minor groove binding. Ethidium molecule is colored green for clarity. Bottom panel: Overlay of X-ray experimental structure (NDB code drbb12) and a 10 μs average structure (green) from the intercalation event from panel B.

The base-pair eversion mechanism has been reported earlier in related works (80–82). Briefly, the ligands explore the minor groove towards an A/T base-pair as early in the simulation as ∼50 ns from the starting positions. Using the dynamic breathing motions of DNA, the ethidium molecule pushes both adenine and thymine nucleobases towards the major groove until the nucleotides flip open towards the major groove. This process involves between ∼100–1000 ns of sampling time and is mainly the oscillation of the ethidium molecule pushing the bases (not the very few ps seen in the earlier cited work of Monaco). The ethidium proceeds to move into the resulting cavity, forming stacking interactions with both base-pairs within the cavity. Minor groove intercalation (Figure 6c) was found in three of the 20 sampled copies; two were found to be in an ApA base step and one in the ApC step. For all three observed cases, the intercalation process was similar: A base-pair eversion mechanism occurs and the everted nucleobases reform their Watson-Crick pairing after an average of ∼2–3 μs. Intercalation always started within the minor groove with the phenanthridine side of the ethidium towards the floor of the minor groove which starts the eversion mechanism and it was an irreversible event whereas if the ethidium only produces base-pair eversion, we found that this event was reversible in all 20 independent copies. Selected frames of the above process showing structural details are depicted in Supplementary Figure S1 and the included molecular graphics movie file in the supporting information. Using the intercalation event on the ApA step, we calculated an average structure over 10 μs of that particular step with the intercalated ethidium and calculated the root mean square deviation with the experimental structure (NDB code drbb12 (15)) which resulted in a value of 0.7 Å which is considered very close agreement. Intercalation of ethidium shifted the helical twist of the GAAC sequence to a value of 31.9°, which is the same value as reported in the experimental crystal (83). In comparison, the twist value of a GAAC simulation with no ethidium molecule has an average of 34.5° (three independent simulation replicas, each ∼15 μs). The last binding mode found was indeed unexpected and was observed only in one case. The ethidium molecule moved throughout the solvent towards the major groove until it reached a CpG step at the middle of the GAAC sequence. The phenanthridine side interacts with N4 of the dC28 then moves toward N4 of the dC8 (on the complementary strand) which produces an increase in helical bend and roll of the base-pairs. This space allows the phenanthridine to slide within the base-pairs, which causes that particular base-step to unwind (Figure 6D).

### ApA base step intercalation is preferred over G/C site from umbrella sampling simulations

We have presented unbiased simulations of two different double-stranded DNA sequences and their interaction with the ethidium molecule. For the DDD system, three independent >93 μs were sampled; showing the ethidium molecule binding at both ends of the terminal base-pairs in a stacking interaction. To reduce this artifact due to the shortness of the DNA sequence, we sampled twenty independent simulations of the GAAC 18-mer, each reaching >27 μs. Both tested systems included four of the ethidium molecules. The GAAC showed the observed stacking interactions at both ends of the termini base-pairs as observed with the DDD. In addition, all the sampled trajectories show the ethidium exploring the minor groove, which lead to either intercalation or base-pair eversion. This latter binding mode was observed to start only when an ethidium molecule is within the minor groove, whereas the intercalation interaction was observed to start both from the major and the minor groove. In order to study the energetic preference of this process, we conducted a series of biased simulations using the umbrella sampling approach. The reaction coordinate used consisted in a distance restraint between the center of mass of the base pairs before and after a manually intercalated ethidium molecule and the mentioned ethidium. One set of simulations started with the ethidium from the major groove and another from the minor groove. Both ApA and GpG steps were employed in both situations to provide some insight regardless of sequence preference.

**Figure 7.**
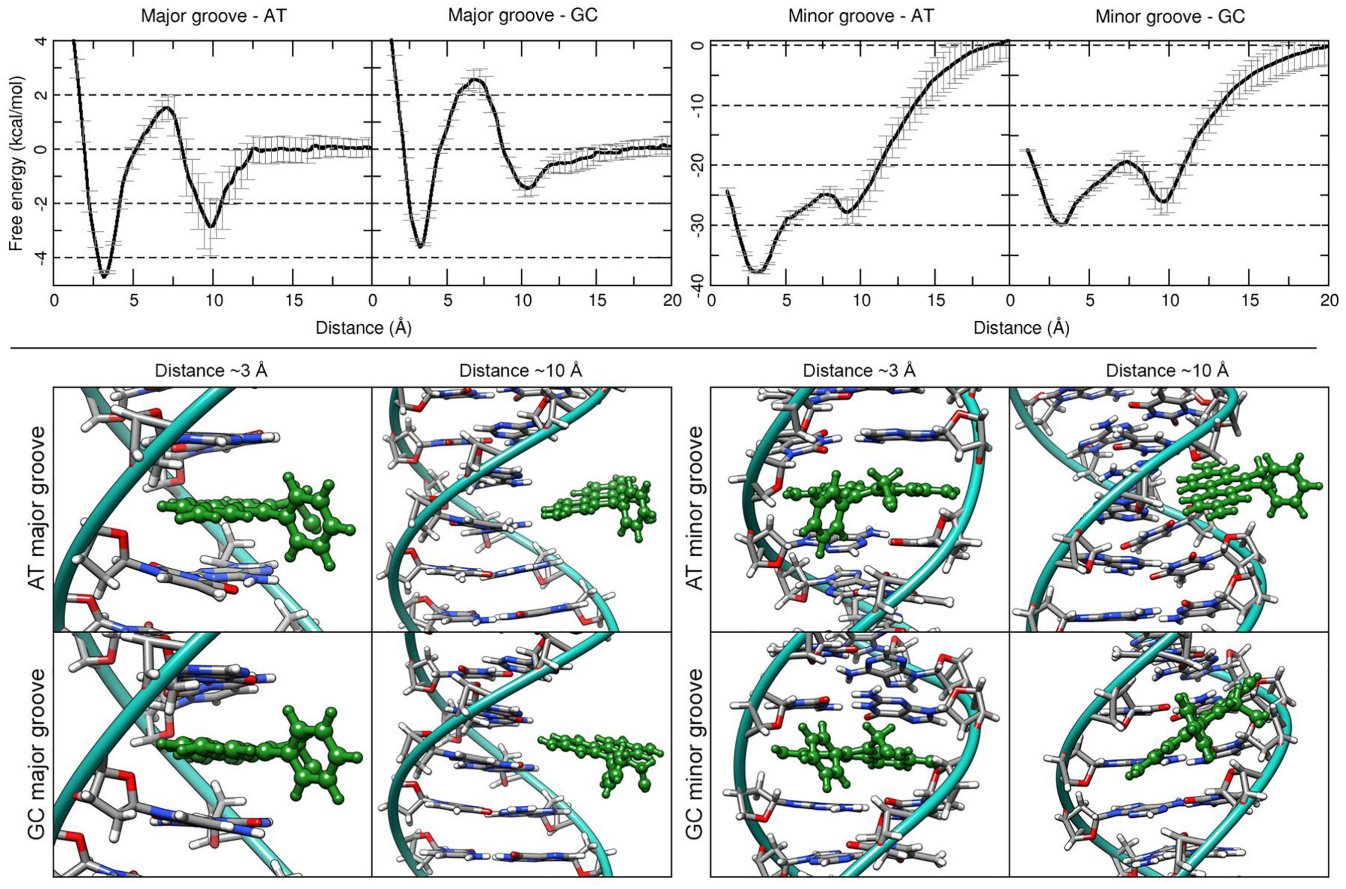
Top row: Free energy profiles from the umbrella sampling simulations using a distance restraint as the reaction coordinate between the center of mass of the DNA (either an ApA or a GpG step) and the center of mass of an ethidium molecule. The profile is the average of five independent runs per window, standard deviation is represented by the error bars. Major and minor groove represents at what side the intercalation was started. Bottom: representative structure at the global (∼3 Å) and the local (∼10 Å) minima. Ethidium molecules are depicted in green for clarity.

Free energy profiles for the umbrella sampling experiments show a preference of the ethidium molecule to bind within ApA steps (–37.1 and –30.0 kcal/mol for ApA and GpG steps, respectively, which is consistent with the trends seen in experiment) and from the minor groove (Figure 7). Binding values for ethidium intercalating from the major groove correspond to –4.3 and –3.5 kcal/mol for ApA and GpG steps, respectively. Major groove intercalation simulations presented a global energy minimum at the 3 Å distance window that corresponds to the phenanthridinium moiety forming stacking interactions with the nucleobases in the intercalation site. A local energy minimum is present at the 10 Å window corresponding with the ethidium leaving the pocket towards the major groove, interacting loosely via hydrogen bonds with the backbone of the DNA (Figure 7, bottom left). Similarly, to the major groove simulations, the minor groove presented a global energy minimum at the ∼2.8 Å window that corresponds to the phenanthridinium rings intercalating within the DNA bases (Figure 7, bottom right). The global minima represent the ethidium molecule interacting with the minor groove of the DNA. In both cases, the 5-ethyl-6-phenyl substituents of the phenanthridinium form a robust network of hydrogen bonds that increase the stabilization of the ligand when intercalating within the minor groove. When in the intercalation pocket, the phenyl ring interacts with O5 from thymine and O2 from cytosine. When in the minor groove, the ethidium molecule forms extensive hydrogen bonds with the atoms present in the floor of the minor groove and with the back-bone of the DNA (84).

### Free energy calculations from unbiased simulations shows a strong preference for intercalation through minor groove binding

To measure the binding energy between the ethidium molecule and the DNA without any bias, we performed simulations with the ethidium manually intercalated between an ApT step and a GpC step using the sequences d(CGCGAATTCGCG) and d(CGCGAGCTCGCG) respectively (refer to Supplementary Figure S2 for a representative snapshot for each system). The ethidium ligand was inserted between these steps both from the major groove and the minor groove and each simulation was run using three independent copies, each copy at least 5 μs of sampling time. Ethidium-DNA interaction through the major groove using every 10th frame from all three independent copies and quasi-harmonic entropy approximation show a binding free energy of –18.3 (±2.1) kcal/mol and –22.8 (±2.5) kcal/mol for ApT and GpC steps respectively. Interaction through the minor groove show binding energy values of –28.0 (±3.2) for the ApT steps and –28.1 (±2.8) kcal/mol for the GpC step. The curvilinear helicoidal coordinates analysis for these four systems is depicted in Supplementary Table S1, which confirms the location of the ethidium ligand within the major and minor grooves for the duration of the simulations. The binding energies from the unbiased simulations with the ethidium manually intercalated into DNA can be compared with the umbrella sampling simulations that show the minor groove interaction being more stable than intercalation through the major groove. One possible explanation to the stability of the DNA–ethidium complex when the ligand is in the minor groove is an increased number of hydrogen-bonds between ethidium and the backbone (84). We tested this hypothesis with a hydrogen-bond population analysis (see Supplementary Table S1 in the Supporting Information) between the DNA and the ethidium ligand using the exact same number of frames for each system (corresponding to 1.2 μs). The main hydrogen-bond interaction is between the protons present in positions 3 and 8 from the amino groups and the O4′ oxygen of the deoxyribose molecule. As can be seen from the data, the mentioned hydrogen-bond is the most populated interaction for all the cases, regardless of the intercalation side. This is due to the position of the amino groups forming the H-bond donors, positioned on each side in a linear manner across the phenanthridinium rings. These allows a close contact with the oxygen from the deoxyribose from both sides of the DNA duplex. It has been reported that the main stabilization interaction between intercalated ethidium and the DNA nucleobases is provided by aromatic stacking interactions, mainly through hydrophobic interactions and dispersion energy (85–87). The short simulations presented by Monaco and collaborators support the idea of ethidium binding to any GXXG site, forming hydrogen bonds with guanine's O6 and N7 centers (40,41). Even though our data with the GAAC sequence do detect the formation of these hydrogen bonds and support the idea of the ethidium molecule exploring the major groove of the DNA, any binding event observed was short lived, with no discernable sequence preference, as seen, for example, when the ethidium ligand is bound within the minor groove (high density population close to the ApA steps as observed in Figure 4).

## CONCLUSION

The simulations presented in this work allowed us to observe naturally occurring intercalation events without any bias or restraint. The minor groove binding showed a two-step mechanistic pathway of interaction which involves the base-pair eversion mechanism as a meta-stable step with the reforming of the Watson–Crick pairing to complete the intercalation whereas our single major groove binding event was driven by the present breathing and dynamics of the DNA duplex. The ethidium molecule co-exists in both an intercalative binding mode and an electrostatic binding mode within the minor groove. This work also stresses the relevance of extended sampling time in molecular dynamics simulations and the use of multiple replicas to increase statistical significance, especially for biomolecular processes. Intercalation of small molecules with nucleic acids is extremely relevant for drug design, since it is one of the main binding modes. We have presented in this work that MD simulations, with the use of the most recent AMBER force fields and methodologies, including the optimized GPU codes which facilitates extended simulation time scales, allows future research on selective drugs targeting nucleic acids by all known binding modes (with the exception of covalent inhibitors).

## DATA AVAILABILITY

Pre-processed trajectories of all the simulations (no solvent molecules, imaged and RMS fit), topology files and analysis scripts are available for download at the URL: https://amber.utah.edu/DNA-dynamics/GAAC-ethidium/

## Supplementary Material

gkab143_Supplemental_Files
