# Supplementary material for: Ethidium bromide interactions with DNA: an exploration of a classic DNA–ligand complex with unbiased molecular dynamics simulations
Source: Nucleic Acids Res. 2021 Mar 25;49(7):3735–47. doi: 10.1093/nar/gkab143 (PMC8053101; doi:10.1093/nar/gkab143)
Supplement: gkab143_Supplemental_Files [file gkab143_supplemental_files.zip › revised-SI-20210203-rgm.docx]

Ethidium bromide interactions with DNA. An exploration of a classic DNA-ligand complex with unbiased molecular dynamics simulations.

Rodrigo Galindo-Murillo and Thomas E. Cheatham, III*

Department of Medicinal Chemistry, College of Pharmacy, University of Utah, 2000 East 30 South Skaggs 306, Salt Lake City, Utah 84112, United States.

**Supplemental Information.**

**Figure S1**. Representative snapshots showing structural details of the formation of the intercalation of ethidium in the GAAC sequence. A) ethidium explores the minor groove with the phenanthridine side towards the floor of the minor groove, B) ethidium pushes an AT base-pair, C) insertion of the phenanthridine planar moiety in the resulting cavity with eversion of a base pair, D) flip back of the adenine nucleobase to the helical DNA center, E) adenine nucleobase in the canonical position stacking with the ethidium molecule and F) reformation of the AT Watson-Crick pairing with the ethidium in intercalation mode. Note that the supporting information also contains a movie highlighting these processes.

**
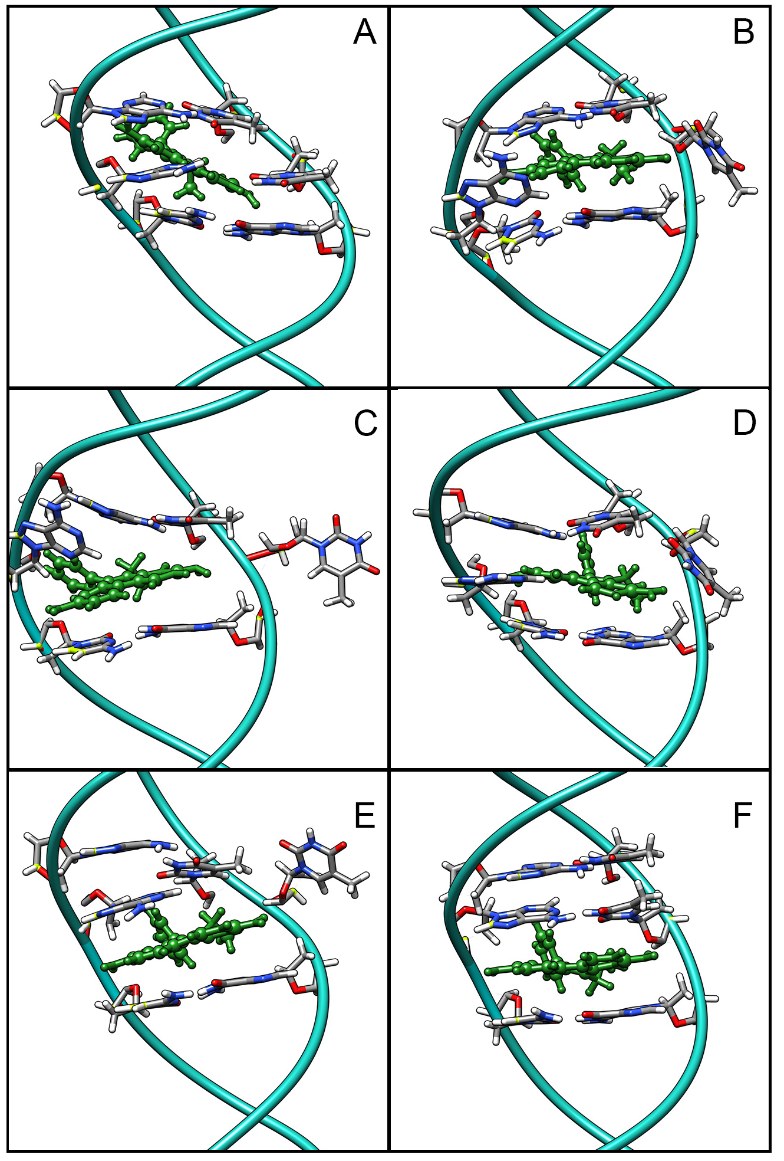
**

**Figure S2**. Top: representative snapshot for a single frame extracted from the intercalation simulations. Left to right: major groove, ApT; major groove GpC; minor groove ApT and minor groove GpC. Ethidium ligand is colored green for clarity. Bottom: one-dimension A, D and R plots of the ethidium ligands. Analysis was performed using the whole trajectory from the three independent copies. The short distance in the A plot between the dotted lines represents the minor groove using the C1’ atoms angular position. The dashed line in the R plot represents the radial position of the phosphorous atoms.


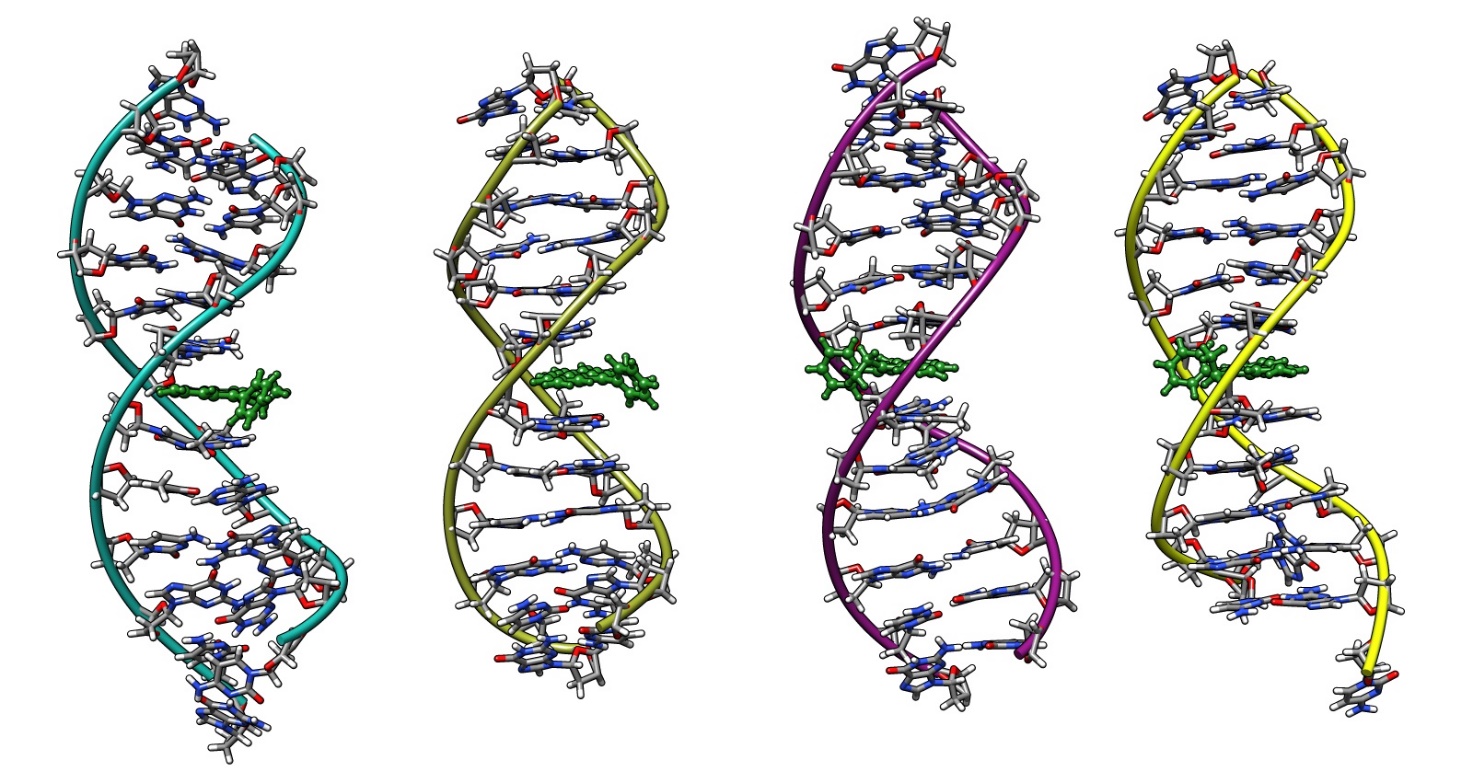


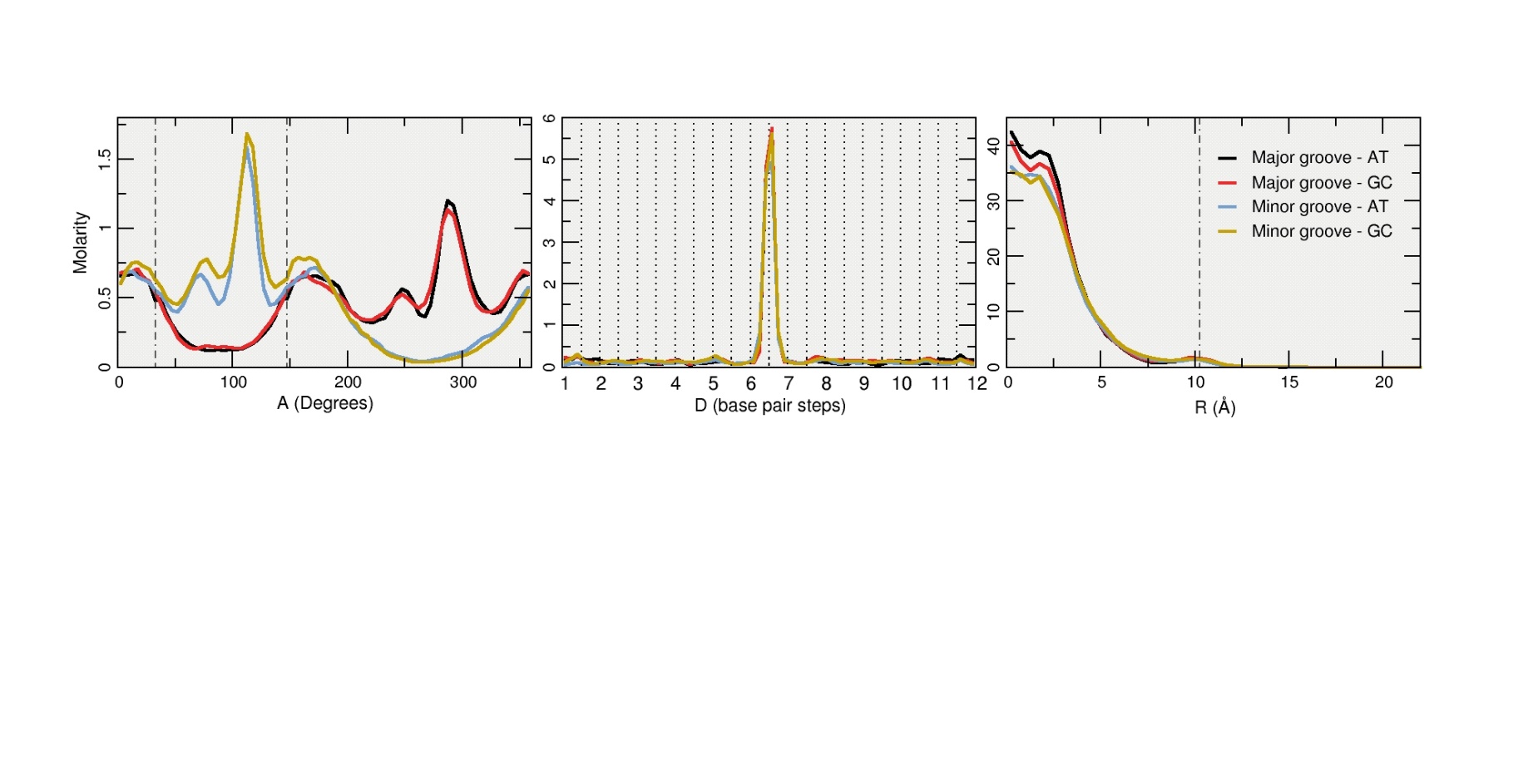


**Table S1.** Hydrogen bond analysis from the unbiased simulations with the manually placed ethidium ligand. Data calculated using the exact same number of frames from all three independent copies. Only significant hydrogen bonds are shown. The atom names for the ethidium here are depicted in the figure on the next page.

| Major groove - AT | | | | |  | | | | | Major groove GC | | | | |
| --- | --- | --- | --- | --- | --- | --- | --- | --- | --- | --- | --- | --- | --- | --- |
| #Acceptor | DonorH | Donor | Frames | Fraction | AvgDist | AvgAng |  | #Acceptor | DonorH | Donor | Frames | Fraction | AvgDist | AvgAng |
| DT_19@O4' | ETH_25@H21 | ETH_25@N23 | 7552 | 0.1247 | 2.8 | 154.1 |  | DC_19@O4' | ETH_25@H22 | ETH_25@N23 | 9367 | 0.1546 | 2.8 | 152.7 |
| DT_19@O4' | ETH_25@H22 | ETH_25@N23 | 6603 | 0.109 | 2.8 | 154.5 |  | DC_19@O4' | ETH_25@H21 | ETH_25@N23 | 8399 | 0.1386 | 2.8 | 152.5 |
| DT_7@O4' | ETH_25@H34 | ETH_25@N | 5976 | 0.0986 | 2.8 | 153.2 |  | DC_7@O4' | ETH_25@H34 | ETH_25@N | 4054 | 0.0669 | 2.8 | 152.3 |
| DT_7@O4' | ETH_25@H33 | ETH_25@N | 5073 | 0.0837 | 2.8 | 153.0 |  | DC_7@O4' | ETH_25@H33 | ETH_25@N | 3428 | 0.0566 | 2.8 | 151.8 |
| DA_6@O3' | ETH_25@H33 | ETH_25@N | 431 | 0.0071 | 2.8 | 144.4 |  | DC_19@O5' | ETH_25@H21 | ETH_25@N23 | 340 | 0.0056 | 2.9 | 149.8 |
| DA_6@O3' | ETH_25@H34 | ETH_25@N | 397 | 0.0066 | 2.8 | 144.3 |  | DG_6@O3' | ETH_25@H33 | ETH_25@N | 302 | 0.005 | 2.8 | 146.6 |
| DT_7@O5' | ETH_25@H33 | ETH_25@N | 126 | 0.0021 | 2.9 | 152.9 |  | DC_19@O5' | ETH_25@H22 | ETH_25@N23 | 276 | 0.0046 | 2.9 | 148.8 |
| DA_18@O3' | ETH_25@H21 | ETH_25@N23 | 121 | 0.002 | 2.8 | 143.6 |  | DC_7@O5' | ETH_25@H34 | ETH_25@N | 273 | 0.0045 | 2.9 | 152.6 |
| DT_7@O5' | ETH_25@H34 | ETH_25@N | 120 | 0.002 | 2.9 | 155.9 |  | DG_6@O3' | ETH_25@H34 | ETH_25@N | 265 | 0.0044 | 2.8 | 148.1 |
| DA_18@O3' | ETH_25@H22 | ETH_25@N23 | 96 | 0.0016 | 2.8 | 145.3 |  | DC_7@O5' | ETH_25@H33 | ETH_25@N | 181 | 0.003 | 2.9 | 155.1 |
| DT_19@O5' | ETH_25@H22 | ETH_25@N23 | 69 | 0.0011 | 2.9 | 150.0 |  | DG_18@O3' | ETH_25@H22 | ETH_25@N23 | 174 | 0.0029 | 2.8 | 146.0 |
| Minor groove - AT | | | | |  | | | | | **Minor groove - GC** | | | | |
| DT_7@O4' | ETH_25@H22 | ETH_25@N23 | 5007 | 0.0827 | 2.8 | 149.5 |  | DC_7@O4' | ETH_25@H22 | ETH_25@N23 | 9122 | 0.1506 | 2.8 | 149.1 |
| DT_7@O4' | ETH_25@H21 | ETH_25@N23 | 3378 | 0.0558 | 2.8 | 149.3 |  | DC_7@O4' | ETH_25@H21 | ETH_25@N23 | 8519 | 0.1406 | 2.8 | 149.1 |
| DT_19@O4' | ETH_25@H33 | ETH_25@N | 2210 | 0.0365 | 2.9 | 150.6 |  | DC_19@O4' | ETH_25@H34 | ETH_25@N | 3790 | 0.0626 | 2.9 | 149.6 |
| DT_19@O4' | ETH_25@H34 | ETH_25@N | 1632 | 0.0269 | 2.9 | 150.6 |  | DC_19@O4' | ETH_25@H33 | ETH_25@N | 3526 | 0.0582 | 2.9 | 149.3 |
| DT_19@O5' | ETH_25@H33 | ETH_25@N | 1297 | 0.0214 | 2.9 | 156.1 |  | DC_7@O5' | ETH_25@H21 | ETH_25@N23 | 759 | 0.0125 | 2.9 | 147.8 |
| DT_7@O5' | ETH_25@H22 | ETH_25@N23 | 1284 | 0.0212 | 2.9 | 151.7 |  | DC_7@O5' | ETH_25@H22 | ETH_25@N23 | 654 | 0.0108 | 2.9 | 148.0 |
| DT_7@O5' | ETH_25@H21 | ETH_25@N23 | 919 | 0.0152 | 2.9 | 151.1 |  | DC_19@O5' | ETH_25@H34 | ETH_25@N | 637 | 0.0105 | 2.9 | 152.2 |
| DT_19@O5' | ETH_25@H34 | ETH_25@N | 864 | 0.0143 | 2.9 | 156.0 |  | DC_19@O5' | ETH_25@H33 | ETH_25@N | 575 | 0.0095 | 2.9 | 152.9 |
| DA_6@O3' | ETH_25@H22 | ETH_25@N23 | 67 | 0.0011 | 2.9 | 144.4 |  |  |  |  |  |  |  |  |

**Ethidium bromide coordinates and charges used in this work in mol2 (TRIPOS) format.**


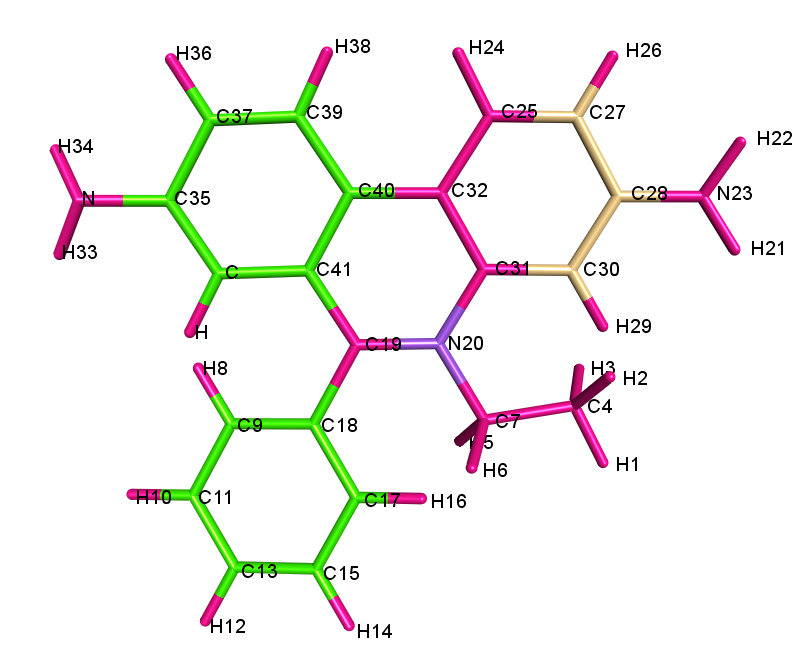


@<TRIPOS>MOLECULE

ETH

44 47 1 0 1

SMALL

USER_CHARGES

@<TRIPOS>ATOM

1 C -1.756380 1.837696 -0.050185 ca 1 ETH -0.3495 ****

2 H -2.699982 1.331229 -0.031406 ha 1 ETH 0.2039 ****

3 C41 -0.552833 1.084275 0.002931 ca 1 ETH 0.0290 ****

4 C40 0.695159 1.722832 -0.020373 ca 1 ETH 0.1064 ****

5 C39 0.700239 3.136392 -0.091825 ca 1 ETH -0.2531 ****

6 H38 1.624373 3.677041 -0.109844 ha 1 ETH 0.1733 ****

7 C37 -0.458994 3.841054 -0.131231 ca 1 ETH -0.0940 ****

8 H36 -0.420928 4.915177 -0.170920 ha 1 ETH 0.1708 ****

9 C35 -1.726887 3.201081 -0.113755 ca 1 ETH 0.3534 ****

10 N -2.871258 3.974022 -0.099916 nh 1 ETH -0.8858 ****

11 H34 -2.805230 4.871653 -0.527606 hn 1 ETH 0.3915 ****

12 H33 -3.721991 3.508925 -0.329741 hn 1 ETH 0.3915 ****

13 C32 1.887265 0.924383 0.031816 cc 1 ETH 0.0695 ****

14 C31 1.789476 -0.467885 0.105762 cc 1 ETH 0.0092 ****

15 C30 2.945890 -1.262404 0.160766 cd 1 ETH -0.2596 ****

16 H29 2.890869 -2.328587 0.198810 ha 1 ETH 0.1838 ****

17 C28 4.200066 -0.688170 0.141291 cd 1 ETH 0.3239 ****

18 C27 4.304925 0.719781 0.065698 cd 1 ETH -0.1440 ****

19 H26 5.276097 1.179209 0.042997 ha 1 ETH 0.1702 ****

20 C25 3.187302 1.484366 0.014375 cc 1 ETH -0.2377 ****

21 H24 3.304404 2.547556 -0.041145 ha 1 ETH 0.1897 ****

22 N23 5.333203 -1.452744 0.144825 nh 1 ETH -0.8693 ****

23 H22 6.192053 -1.013223 0.389258 hn 1 ETH 0.4006 ****

24 H21 5.265233 -2.400008 0.442278 hn 1 ETH 0.4006 ****

25 N20 0.512741 -1.058887 0.130651 na 1 ETH 0.0189 ****

26 C19 -0.592838 -0.336844 0.081358 cc 1 ETH 0.0847 ****

27 C18 -1.926626 -1.021328 0.115532 ca 1 ETH 0.1036 ****

28 C17 -2.560133 -1.235543 1.334210 ca 1 ETH -0.1120 ****

29 H16 -2.086221 -0.933746 2.251432 ha 1 ETH 0.1379 ****

30 C15 -3.806604 -1.837456 1.365163 ca 1 ETH -0.1893 ****

31 H14 -4.293242 -2.005527 2.308029 ha 1 ETH 0.1690 ****

32 C13 -4.425886 -2.215933 0.183434 ca 1 ETH -0.0545 ****

33 H12 -5.393823 -2.681341 0.210072 ha 1 ETH 0.1478 ****

34 C11 -3.800603 -1.987539 -1.031426 ca 1 ETH -0.1893 ****

35 H10 -4.282274 -2.271100 -1.948896 ha 1 ETH 0.1690 ****

36 C9 -2.551896 -1.387053 -1.068722 ca 1 ETH -0.1120 ****

37 H8 -2.076877 -1.196532 -2.014485 ha 1 ETH 0.1379 ****

38 C7 0.431782 -2.547343 0.209812 c3 1 ETH 0.0190 ****

39 H6 1.156477 -2.863271 0.940755 h1 1 ETH 0.0650 ****

40 H5 -0.533607 -2.798776 0.604108 h1 1 ETH 0.0650 ****

41 C4 0.639759 -3.223411 -1.140958 c3 1 ETH -0.0424 ****

42 H3 -0.164390 -2.977359 -1.822414 hc 1 ETH 0.0358 ****

43 H2 1.576389 -2.945737 -1.605428 hc 1 ETH 0.0358 ****

44 H1 0.636771 -4.298034 -0.996801 hc 1 ETH 0.0358 ****

@<TRIPOS>BOND

1 1 2 1

2 1 3 1

3 1 9 1

4 3 4 1

5 3 26 1

6 4 5 1

7 4 13 1

8 5 6 1

9 5 7 1

10 7 8 1

11 7 9 1

12 9 10 1

13 10 11 1

14 10 12 1

15 13 14 1

16 13 20 1

17 14 15 1

18 14 25 1

19 15 16 1

20 15 17 1

21 17 18 1

22 17 22 1

23 18 19 1

24 18 20 1

25 20 21 1

26 22 23 1

27 22 24 1

28 25 26 1

29 25 38 1

30 26 27 1

31 27 28 1

32 27 36 1

33 28 29 1

34 28 30 1

35 30 31 1

36 30 32 1

37 32 33 1

38 32 34 1

39 34 35 1

40 34 36 1

41 36 37 1

42 38 39 1

43 38 40 1

44 38 41 1

45 41 42 1

46 41 43 1

47 41 44 1

@<TRIPOS>SUBSTRUCTURE

1 ETH 1 **** 0 **** ****
